# Bacteriophages and the Microbiome in Dermatology: The Role of the Phageome and a Potential Therapeutic Strategy

**DOI:** 10.3390/ijms24032695

**Published:** 2023-01-31

**Authors:** Nicole Natarelli, Nimrit Gahoonia, Raja K. Sivamani

**Affiliations:** 1 Morsani College of Medicine, University of South Florida, 560 Channelside Drive, Tampa, FL 33602, USA; 2 College of Osteopathic Medicine, Touro University, 1310 Club Drive, Vallejo, CA 94592, USA; 3 Integrative Skin Science and Research, 1495 River Park Drive, Sacramento, CA 95815, USA; 4 Pacific Skin Institute, 1495 River Park Drive, Sacramento, CA 95815, USA; 5 College of Medicine, California Northstate University, 9700 W Taron Dr, Elk Grove, CA 95757, USA; 6 Department of Dermatology, University of California-Davis, 3301 C Street #1300, Sacramento, CA 95816, USA

**Keywords:** bacteriophage, phage, acne, psoriasis, atopic dermatitis, dysbiosis, microbiome, gut, skin

## Abstract

Bacteriophages, also known as phages, are viruses that selectively target and infect bacteria. In addition to bacterial dysbiosis, dermatologic conditions such as acne, psoriasis, and atopic dermatitis are characterized by a relative reduction in the abundance of phages and the overgrowth of the corresponding bacteria. Phages often exhibit high specificity for their targeted bacteria, making phage-replacement therapy a promising therapeutic strategy for the control of pathogenic bacteria in dermatologic disease. Novel therapeutic strategies regulating pathogenic bacteria are especially necessary in light of growing antibiotic resistance. In this review, we aimed to review the medical literature assessing phage dysbiosis and therapeutic trials in dermatology. Ultimately, studies have depicted promising results for the treatment of acne, psoriasis, and atopic dermatitis but are limited by low sample sizes and the omission of control groups in some trials. Additional work is necessary to validate the efficacy depicted in proof-of-concept trials and to further determine optimal treatment vehicles, administration mechanisms, and dosing schedules. This review provides the necessary framework for the assessment of phage efficacy in future trials.

## 1. Introduction

Bacteriophages, also known as phages, are a type of virus that selectively target and infect bacteria [1]. Phages are able to propagate among bacteria and they are very specific to the type of bacteria they infect. Although they do not infect human cells, they do have a profound impact on the bacteria that exist within and on humans. For instance, many highly virulent bacteria such as *Corynebacterium diphtheriae* have contracted the ability to produce their pathogenic toxin from genetic material acquired by phages [1]. Additionally, phages have been involved with the spread and transduction of antibiotic resistance genes among bacteria. Although some temperate phages have been responsible for the emergence of pathogenic bacterial strains, lytic phages have been used to prevent and treat human infections for decades; as such, phage maintenance of the human gut microbiome continues to be of interest. The literature has well established the importance of the gut and skin microbiome in the maintenance of overall health and skin. The gut microbiome, composed of an array of microbes, is vital to the proper digestion of foods, in the defense against disease-causing pathogens, secondary metabolite production, and immune regulation [2]. Since phages proliferate among bacteria, a more recent concept of the importance of a phageome has been proposed. A phageome is defined as the community of phages that exist in a certain environment [3]. It has been theorized that the phageome has a substantial effect on regulating the microbiome [4], whether it is on the skin or in the gut. A balanced microbiome is thought to be dynamically controlled by phages. Lytic phages prevent the overgrowth of harmful bacteria by directly causing lysis, and temperate phages are believed to promote symbiotic bacteria in the gut by providing genes that allow them to survive [5]. This intricate balance is fundamental to the functioning of a healthy microbiome. Deviations from a balanced microbiome have been implicated in many inflammatory dermatologic conditions including atopic dermatitis, psoriasis, and acne vulgaris [6]. Among these conditions, many are associated with gut dysbioses, which will not be discussed in this review.

To date, the literature has extensively researched and reviewed the strong association between skin health and a healthy skin microbiome. However, the role of the phageome in regards to skin health has not been as substantially explained. Currently, among the studies that have researched the skin phageome, many have highlighted the lack of a diverse viral database that has been a prominent limiting factor for fully assessing the types of bacteriophages present on the skin [7,8]. Regardless of this limitation, the importance of the skin phageome to skin health has still been supported. For instance, it has been shown that the viral diversity present in chronic wounds increases compared to healthy skin [9]. Another example is a study conducted to assess the differences within the skin phageome in normal skin vs. lesional skin in psoriasis patients that reported differences in the species of phages that were most abundant in each group [10]. Although the significance of the findings from these studies is not clear for clinical intervention, it is likely there is some role of the skin phageome in influencing cutaneous microbiome and health. Consequently, this has inspired research studies to investigate the possibility of phage therapy as a treatment modality for cutaneous conditions. 

The unique properties of phages have contributed to the recent re-emergence of interest in phage therapy. Phage therapy involves the administration of phages that selectively target pathogenic bacteria in order to alleviate associated health conditions [11]. Although phage therapy is largely still in its infancy, it holds tremendous promise for acting as an antimicrobial treatment and restoring healthy skin and gut microbiomes. In this review, we summarized the current advances utilizing phage therapy for targeting various dermatologic conditions. 

## 2. Phage and Dermatologic Disease

**Figure 1:**
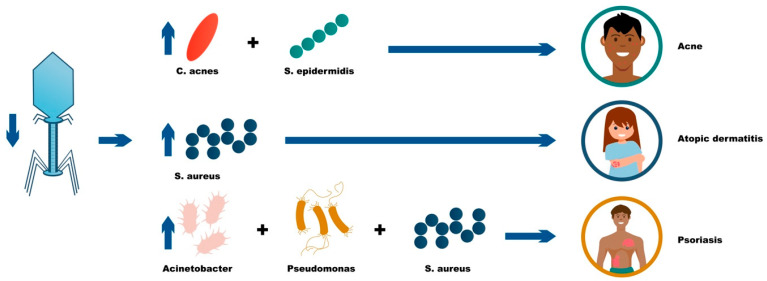
The influence of phage deficiency in acne, atopic dermatitis, and psoriasis.

Previous investigations have assessed microbial and phage compositions among patients with dermatologic disease. Reduced phage concentrations are typically associated with an abundance of the corresponding bacteria, depicting the capacity of phages to suppress their host bacteria [10]. Figure 1 depicts the increased pathologic bacteria corresponding with reduced phage concentrations characteristic of dermatologic disease. Phage supplementation, either orally or topically, may function to replenish phages with a subsequent reduction in the corresponding pathogenic bacteria. Researchers have assessed potential phage-based therapeutic strategies for a variety of dermatologic conditions, including psoriasis, acne, and atopic dermatitis. In addition, few phage therapy patient testimonials are available online among patients with hidradenitis suppurativa (HS), although no studies to our knowledge have directly assessed phage therapy efficacy for HS. 

### 2.1. Psoriasis

Affecting almost 3% of the population in the United States, psoriasis is a chronic inflammatory disease characterized by plaques and scaly lesions [12]. Although the etiology is not fully understood, psoriasis is characterized by keratinocyte hyperproliferationa [13] and an increased secretion of inflammatory cytokines including interleukin (IL)-17, IL-21, IL-23, and tumor necrosis factor (TNF)-alpha [14]. In addition, many studies have identified gut [15] and skin [16] microbial dysbioses in patients with psoriasis. For example, psoriasis severity is correlated with enterotoxin production by some *Staphylococcus aureus* strains. Furthermore, bacterial alpha diversity has been shown to be reduced in psoriatic lesional skin than in healthy control skin [14,15]. As phage composition is intricately related to microbial composition, researchers have investigated the phage component of the cutaneous microbiome among patients with psoriasis [10]. 

#### 2.1.1. Phage and Microbial Dysbiosis

In a 2020 study, psoriatic lesional skin was obtained from the elbow, forearm, knee, and scalp of sixteen patients with psoriasis [10]. Healthy skin samples from matched locations were obtained from contralateral non-lesional skin and matched family controls. Ultimately, 27 lesional skin samples and 54 healthy skin samples were analyzed for their phage and bacterial components.

The authors noted a significantly greater abundance of the top ten most-abundant phage species in the healthy skin compared to lesional skin [10]. *Acinetobacter* phage Presley, *Salmonella* phage vB_SenS-Ent2, and *Bacillus* phage SP-10 were the most differentially abundant phages. In addition, the authors observed a significantly greater bacterial alpha diversity in the healthy skin than the lesional skin (*p* < 0.001) [10], similar to observations of prior studies [17,18]. 

The authors further examined two phage species, *Acinetobacter* phage Presley and *Pseudomonas* phage O4, and their corresponding bacterial genera. While the family control skin depicted the highest abundance of these two phages, an intermediate and low abundance was demonstrated by the contralateral non-lesional skin and lesional skin, respectively (*p* = 0.02 for each phage) [10]. In addition, those with a greater phage abundance had a significantly lower abundance of corresponding host bacteria (*p* = 0.03 for *Acinetobacter*; *p* < 0.001 for *Pseudomonas*), demonstrating the capacity of phages to suppress the abundance of their host bacteria [10]. This finding strongly suggested that the connection between phage communities and analogous cutaneous bacterial communities may be important in the cutaneous microbiome with psoriasis. 

#### 2.1.2. Potential Therapeutic Strategies 

The results of the study conducted by Wang et al. [10] support the incorporation of phages as ingredients in topical approaches for the treatment of psoriasis, thereby replenishing phages characterized by a low abundance in lesional skin and potentially correcting cutaneous bacterial dysbiosis. Prior studies observed promising results with phage therapy for skin conditions caused by opportunistic *Acinetobacter baumannii* and *Pseudomonas aeruginosa*, such as wound infections. A 2012 study found that the density of *P. aeruginosa* decreased in the presence of phages in human skin (ex vivo), suggesting that phage application can effectively alter and correct microbiota compositions [19]. In addition, a 2016 study observed a decreased bioburden and infection-associated morbidity in an infected-wound murine model following the consumption of a therapeutic cocktail composed of five phages [20]. These efficacious results, coupled with the phage and microbiota dysbiosis in psoriatic lesion skin described by Wang et al. [10], support future research assessing the therapeutic potential of a phage-based cocktail for the management of psoriasis. 

Interestingly, the oral consumption of Ganga river water, reported to contain over two-hundred isolates of phages, demonstrated clinical benefits in psoriatic patients who were previously refractory to prescription medication [21]. Patients with chronic psoriasis underwent two Ganga water protocols: Ganga water was first consumed for two weeks followed by two weeks of rest and was then consumed for four weeks followed by four weeks of rest. Although twenty patients participated in the first Ganga water protocol, only twelve patients participated in the second protocol due to an eight patient drop-out (three of which observed no benefit during the initial protocol). However, only eight patients were included in the final analysis due to loss at follow-up. 

A Likert Scale from 1–10 was used to categorize the severity of psoriatic lesions. Following the first treatment protocol, the subjects achieved a median improvement of 2.5, although with relapse during the rest period [21]. Similarly, the subjects achieved a median improvement of 2.5 following the second treatment protocol. In contrast to the first treatment protocol, however, relapse did not occur following one month of water consumption followed by one month of rest. The authors thereby concluded that Ganga water may be used for the therapeutic management of psoriasis with a consumption of at least four weeks [21]. Future research is necessary to determine the optimal phage cocktail recipe and the optimal phage cocktail protocol for the treatment of psoriasis. 

In addition, the phage display of anti-inflammatory peptides has been evaluated for psoriasis-like lesions in mice [13]. Phage display refers to the introduction of a deoxyribonucleic acid (DNA) sequence within a functional viral gene with a resulting presentation of exogenous peptides on the capsid surface of phages [22]. Bounded phage-peptides with interferon (IFN)- α/β signaling inhibition were administered intradermally in the back of mice with either imiquimod-induced psoriasis or 12-O-tetradecanoyl phorbol-13-acetate-induced psoriasis [13]. The authors observed reduced skin thickness, redness, and acanthosis and decreased production of IL-17A and TNF-α in the psoriatic murine skin. The results of these studies suggested that phage cocktail consumption and display may be promising therapeutic strategies for the treatment of psoriasis. 

### 2.2. Acne 

Affecting an estimated 85% of individuals within their lifetime [23], acne vulgaris is an inflammatory disorder of the pilosebaceous unit, developing from increased sebum production and hyperkeratinization with the resulting clogging of hair follicles [24]. Phenotypic signs include closed or open comedones, pustules, papules, and deep nodules [25]. While hormones, diet, and certain medications can contribute to acne, sebum and hyperkeratinization are believed to stimulate *Cutibacterium acnes* proliferation, further contributing to inflammation and worsening acne severity [24]. Although *C. acnes* has long been regarded to contribute to the pathogenesis of acne, recent work has highlighted other bacteria such as *Staphylococcus epidermidis* [26]. Nevertheless, topical and oral antibiotics are still among the first-line options for the treatment of acne vulgaris. 

#### 2.2.1. Phage and Microbial Dysbiosis

Cutaneous and gastrointestinal microbial dysbioses associated with acne have been previously reported. Acne inflammatory lesions are associated with an overabundance of certain strains of *C. acnes* and a reduced alpha diversity in comparison to non-inflammatory lesions of acne patients and healthy control subjects [27]. Similarly, a microbial analysis of twenty-six subjects revealed *Proteobacteria* and *Firmicutes* overabundance and a reduction in the abundance of *Actinobacteria* [26]. Comedone, papule, and pustule surfaces were characterized by a significantly greater abundance of *Staphylococci* compared to non-lesional skin, and the proportions directly increased with acne severity. 

In addition to bacterial dysbiosis between healthy skin and that of acne patients, differences in *C. acnes* phage abundance have been observed. A 2016 study found a greater relative abundance of *C. acnes* phages in healthy skin compared to the matched lesional skin of acne patients (*p* = 0.05) [28], thereby associating phage deficiency with acne. Interestingly, the authors also observed a direct relationship between *C. acnes* phage abundance and age, suggesting phage abundance may contribute to lower acne vulgaris prevalence among aging individuals. Table 1 summarizes the phage dysbiosis observed in both psoriasis and acne [10,28].

Increased *C. acnes* bacterial abundance coupled with decreased *C. acnes* phage abundance in acne patients provides a theoretical framework for the therapeutic replenishment of *C. acnes* phages. However, it is necessary to note that significant gene conservation has been observed among *C. acnes* phages, despite the great diversity observed in essentially all other phage populations [29]. *C. acnes* has evolved strategies to adapt to its cutaneous environment and survive host defenses, such as lipolytic activity, pH regulatory mechanisms, biofilm formation, and the potential activation of dormancy. Such adaptation has resulted in a reduced bacterial diversity, which may have contributed to the observed conservation among *C. acnes*-infecting phage genomes [30]. While beneficial in that phages depict a broad activity against *C. acnes* strains, phage homogeneity poses a therapeutic challenge in that phage host receptor mutations may rapidly confer broad resistance [30]. Still, phage-based therapy is promising, as the bacterial species specificity of phages may allow a therapeutic reduction in pathological strains without affecting commensal bacteria [24]. Furthermore, the ability of phages to penetrate biofilms, a potential virulence factor of *C. acnes*, allows phage-based therapy to be promising for future clinical studies in acne. 

#### 2.2.2. Potential Therapeutic Strategies 

A 2019 study assessed the effect of dorsal bacteriophage injection on *C. acnes*-induced inflammation in murine models [31]. Three groups of mice received an injection of human-isolated *C. acnes* suspension. While group A received no additional intervention (control), group B received an additional injection with *C. acnes* at four weeks, and group C received *C. acnes* and bacteriophages at four weeks. A decreased epidermal thickness and a decreased number and size of microcomedone-like cysts were observed in groups B and C compared to the control group. In addition, the inflammatory nodule size decreased to the greatest extent in group C, depicting the potential efficacy of bacteriophages to reduce *C. acnes*-induced inflammatory nodules [31]. 

In addition, a 2021 study assessed the effect of lytic bacteriophage treatment on multi-drug-resistant *C. acnes* infection in mice, as the antibiotic resistance of *C. acnes* is growing and poses a challenge for conventional acne treatment [32]. Mice were bilaterally injected with *C. acnes* followed by phage injection on one side. The *C. acnes* injection resulted in bilateral inflammatory nodules, although the authors observed a significant reduction in inflammatory lesions on the side injected with the phage. In addition, a significantly decreased expression of the inflammatory marker IL-1β and the apoptotic marker caspase-3 was observed with phage therapy [32]. 

In addition, the authors assessed the phage activity of different phage formulations in hydroxyethyl cellulose (HEC) cream [32]. Full lytic capacity was retained with 0.5% phage HEC cream after 180 days when stored at four degrees Celsius regardless of light protection or at twenty-five degrees Celsius in the dark. However, the storage of 1% phage HEC at either four or twenty-five degrees with light exposure resulted in a loss of lytic capacity by seventy days. The 1% formulation retained its lytic capacity upon storage in the dark [32]. These results depicted the ability to create phage-containing cream formulations for the potential treatment of *C. acnes*-associated inflammatory lesions, although proper storage is necessary for retaining the lytic activity. 

Similarly, a 2016 study isolated ten phages capable of *C. acnes* lysis from human skin microflora and formulated them into a cetomacrogol aqueous cream (2.5 × 10^8^ plaque-forming units (PFU) per gram) [33]. A retained lysis activity was observed for at least ninety days upon storage at four degrees Celsius. The successful creation of a semi-solid phage formulation with *C. acnes* lysis activity marked a necessary step in creating a topical phage-based acne therapy targeting pathogenic *C. acnes*. However, the formulation was not directly assessed in human subjects. 

Whereas the prior studies assessed the effect of phage therapy in the reduction in *C. acnes*-associated inflammatory nodules in murine models, an additional study evaluated the efficacy and safety of a topical phage formulation among human subjects diagnosed with mild-to-moderate acne [24]. The authors created a three-phage cocktail, BX001, using selected phages shown to have high specificity for *C. acnes* with low susceptibility of commensal species. The BX001 cocktail was formulated into an aqueous topical gel. Ex vivo analysis on human skin depicted no irritation effects at any of the studied concentrations, leading the researchers to evaluate the formulations among seventy-five female acne patients stratified into three groups: control, low dose, and high dose. Those receiving the high-dose topical formulation depicted a significantly reduced *C. acnes* bacterial load compared to vehicle control at day 35 (*p* = 0.036), although a bacterial reduction was not observed in the low-dose group [24]. While both doses were well tolerated, the results suggested a dose-dependent reduction in *C. acnes* bacterial load upon the topical application of the phage. However, the authors did not assess the effect of the topical gel on clinical measures of acne, such as inflammatory lesion count or size. Additional research is necessary to assess the clinical efficacy of oral, topical, or intradermal phage formulations among human subjects with acne, although the described studies depict phage replacement to be a promising therapeutic strategy. 

### 2.3. Atopic Dermatitis

Atopic dermatitis (AD) is a chronic inflammatory dermatologic condition commonly present during childhood. Clinically, it presents with patches of intense dryness, scaling, erythema, and pruritus that usually affects the face, neck, scalp, and flexor surfaces of the skin [34]. Typically, these rashes follow a relapsing and remitting course which can significantly affect quality of life. The etiology of AD is complex and multifactorial, but a significant contributor to the pathogenesis is considered to be the colonization and overgrowth of *Staphylococcus aureus* [35]. The flares of patients with AD have shown to incur a shift in the skin microbiome leading to decreased numbers of bacteria that typically suppress *S. aureus* growth [35]. 

### 2.4. Phage Therapy in Atopic Dermatitis

A study conducted in 2020 on atopic mouse models found promising results for the use of a phage in controlling *S. aureus* growth on the skin [36]. The study isolated the phage SaGU1 from Japan. SaGU1 is unique in that it infects only *S. aureus* bacteria and does not disrupt the beneficial *Staphylococcus epidermidis* bacteria present on skin. The in vitro experiment involved assessing the effects of *S. aureus* growth in the presence of SaGU1. During the first 9–13 h, *S. aureus* levels began to decline in the presence of SaGU1; however, they gradually began to increase after 14 h. This finding suggested that it was probable that *S. aureus* began to become phage-resistant. A separate experiment involved determining the effects of SaGU1 on *S. epidermidis* growth, and the results showed that SaGU1 did not affect its growth. Interestingly, the researchers found that when both SaGU1 and *S. epidermidis* were supplied to *S. aureus* cultures, the regrowth of *S. aureus* after 14 h did not occur, and the levels remained suppressed. Therefore, a combination therapy of the phage SaGU1 and *S. epidermidis* may be beneficial in controlling *S. aureus* overgrowth. 

During the in vivo phase of the study, atopic mouse skin was treated individually with *S. epidermidis* or SaGU1 and in combination [36]. Each treatment approach showed statistically significant decreases in *S. aureus* growth on the skin of atopic mice. However, when the combination therapy was compared to the phage SaGU1 treatment alone, there was no statistically significant difference in terms of effectiveness on *S. aureus* growth suppression. This suggested that phage therapy alone may be more effective in vivo than in vitro. Overall, these results are extremely promising for the field of phage therapy in treating AD. Although much more intensive research must be performed to determine if phage therapy is clinically useful, this study provided a unique treatment approach that warrants more investigation. 

## 3. Considerations of Phage Therapy 

Phage-based therapeutic strategies appear promising for the treatment of a variety of dermatologic conditions. There are many theoretical attributes of bacteriophages that make them ideal therapeutic candidates for reducing the presence of pathogenic bacteria implicated in disease: phages can be bactericidal, increase in number over time, minimally disturb commensal microflora, depict efficacy against antibiotic-sensitive and antibiotic-resistant bacteria, can potentially disrupt bacterial biofilms, and exhibit low toxicity [37]. However, there are a few limitations and considerations necessary to note in order to best optimize potential phage therapy. 

### 3.1. Phage Characteristics 

Certain phage characteristics predict their therapeutic success. First, in order to allow optimal bacterial lysis, virulent phages must be selected in contrast to temperate phages. Similarly, phages characterized by a poor killing potential against desired bacteria are sub-optimal for therapy. Toxin-carrying phages should be avoided in addition to those depicting a high transduction potential [37]. Lastly, those depicting poor pharmacokinetics, including absorption, distribution, and survival, should be omitted in therapy. 

### 3.2. Other Factors Mediating Treatment Efficacy 

Additional factors mediate phage-based treatment efficacy, including the phage-to-target bacteria ratio, environmental conditions, accessibility to target bacteria, administration mechanisms, and bacterial resistance [38]. First, although phages can increase in number over the duration of therapy, studies have found that passive therapy, in which sufficient phages are added to lyse all the targeted bacteria, is more effective than active therapy, in which phage replication is required in order to reach concentrations sufficient to lyse all the bacteria. However, passive therapy requires a very high multiplicity of infection, which is further complicated by the immunogenic properties of phages. In addition, studies have found multiple phage doses to be more effective than one single dose [38]. 

Secondly, pH has been shown to affect phage titers, with decreasing pH associated with reduced titers. In addition, phages cannot freely diffuse across membranes and thus require a delivery method to reach target cells and bacteria. Administration techniques include oral, topical, intraperitoneal, intravenous, and intranasal administration [38]. However, stomach acid can negatively impact phage survival, and thus oral administration is often more effective in combination with antacids [38], or it may be more effective if delivered in an encapsulated form. Furthermore, the vehicle in which phages are suspended can impact their accessibility to target bacteria. For example, a 2021 study evaluating phage stability in different solutions observed different preserved phage infectivity in different buffer and infusion solutions [39]. Dulbecco’s phosphate-buffered saline without calcium and magnesium demonstrated the greatest preserved infectivity, although phage-specific differences were observed in each solution. In contrast, a 5% glucose solution demonstrated the greatest negative impact on phage infectivity. Similarly, the authors observed variable phage inactivation for different phages and different products conventionally used for burn wound treatment [40]. In general, highly acidic products had the most adverse effect on phage stability. In addition, it is unclear whether pathogens can effectively access intracellular pathogens, thereby potentially mediating their efficacy in the treatment of diseases caused by intracellular bacteria [38]. Based on these limitations, it is recommended to use an appropriate phage concentration while considering the replicative and survival characteristics of particular phages in specific environmental conditions. Phage stability should be tested in real environmental conditions [38]. 

Lastly, experimental data has demonstrated the presence of phage resistance in up to 80% of studies targeting the intestinal microbiome [41]. Bacterial surface receptors required for phage attachment can be mutated [30] in addition to other bacterial surface components such as lipopolysaccharides, outer membrane proteins, capsules, and appendices [41]. Phage-resistant bacteria can additionally arise due to restriction modification systems and adaptive immunity via the CRISPR-Cas system [41]. However, bacteria often develop resistance to phages at a 10-fold lower rate in comparison to antibiotics, and new phages can be administered to target resistant strains [38], including lab-trained phages and phage cocktail combinations. For example, a 2020 study developed a phage cocktail that was effective against *Pseudomonas aeruginosa* with resistant O-antigen deletion mutants [42]. The phage employed against the resistant bacteria alternatively used core lipopolysaccharides as a receptor rather than the O-antigen. However, five days following the use of a novel five-phage cocktail, resistant variants were noted once again due to wyz and migA mutations. 

Ultimately, phage therapy is not without limitations, including the development of resistance, environmental factors, and the necessary consideration of the optimal administration mechanism. Perhaps such limitations have contributed to the fact that only a few countries have accepted the use of therapeutic phages in humans thus far [30]. However, phage-based therapeutic strategies appear promising for the treatment of various dermatology conditions, although future research is necessary to determine the optimal phage recipes, administration methods, doses, and treatment frequency for particular purposes. 

## 4. Conclusions 

Bacteriophage dysbiosis has been observed on the lesional skin of psoriasis and acne patients. Experimental confirmation of the suppressing capacity of phages on their corresponding bacteria suggests that phage dysbiosis may contribute to the microbial dysbiosis characteristics of each dermatologic condition. In addition, phage deficiency with associated bacterial abundance suggests that phage replacement, either topically or orally via phage “cocktails,” may be an effective therapeutic strategy to mitigate the phenotypic signs and symptoms of disease. Few studies assessing phage-based therapies have been promising for the treatment of psoriasis, acne, and atopic dermatitis, both in murine models and human subjects. However, the current evidence is limited by low sample sizes and the omission of controls in some cases. Future research is necessary to assess the efficacy of phage replacement in large scale studies in addition to determining the optimal treatment vehicles, administration mechanisms, and dosing for particular purposes. 

## Figures and Tables

**Table 1:** Relative reduction in phages associated with dermatologic conditions.

Diagnosis	Reduced Phages in Disease
Psoriasis [10]	*Acinetobacter* phage Presley, *Bacillus* phage SP-10, *Bordetella* virus BPP1, *Burkholderia* virus BcepC6B, *Enterobacteria* phage Sfl, *Haemophilus* phage Aaphi23, *Mycobacterium* phage DrDrey, *Pseudomonas* phage O4, *Rhodoferax* phage P2621B, *Salmonella* phage vB_SenS-Ent2
Acne [28]	*Cutibacterium acnes* phage

## Data Availability

No new data was generated for this review but studies were reviewed from publicly available databases.

## Abbreviations

AD	Atopic dermatitis
DNA	Deoxyribonucleic acid
HEC	Hydroxyethyl cellulose
HS	Hidradenitis suppurativa
IFN	Interferon
IL	Interleukin
OTU	Operational taxonomic unit
PFU	Plaque-forming units
TNF	Tumor necrosis factor
ZO	Tight junction protein 1

## References

[1] Kasman L.M., Porter L.D. Bacteriophages. In StatPearls; StatPearls Publishing: 2022. http://www.ncbi.nlm.nih.gov/books/NBK493185/.

[2] Valdes A.M., Walter J., Segal E., Spector T.D. (2018). Role of the gut microbiota in nutrition and health. BMJ.

[3] Ma Y., You X., Mai G., Tokuyasu T., Liu C. (2018). A human gut phage catalog correlates the gut phageome with type 2 diabetes. Microbiome.

[4] Townsend E.M., Kelly L., Muscatt G., Box J.D., Hargraves N., Lilley D., Jameson E. (2021). The Human Gut Phageome: Origins and Roles in the Human Gut Microbiome. Front. Cell. Infect. Microbiol..

[5] Zuppi M., Hendrickson H.L., O’Sullivan J.M., Vatanen T. (2021). Phages in the Gut Ecosystem. Front. Cell. Infect. Microbiol..

[6] De Pessemier B., Grine L., Debaere M., Maes A., Paetzold B., Callewaert C. (2021). Gut–Skin Axis: Current Knowledge of the Interrelationship between Microbial Dysbiosis and Skin Conditions. Microorganisms.

[7] Oh J., Byrd A.L., Park M., Kong H.H., Segre J.A. (2016). Temporal stability of the human skin microbiome. Cell.

[8] Hannigan G.D., Meisel J.S., Tyldsley A.S., Zheng Q., Hodkinson B.P., SanMiguel A.J., Minot S., Bushman F.D., Grice E.A. (2015). The human skin double-stranded DNA Virome: Topographical and temporal diversity, genetic enrichment, and dynamic associations with the host microbiome. mBio.

[9] Verbanic S., Deacon J.M., Chen I.A. (2022). The chronic wound phageome: Phage diversity and associations with wounds and healing outcomes. Microbiol. Spectr..

[10] Wang H., Chan H.H., Ni M.Y., Lam W.W., Chan W.M.M., Pang H. (2020). Bacteriophage of the Skin Microbiome in Patients with Psoriasis and Healthy Family Controls. J. Investig. Dermatol..

[11] Brives C., Pourraz J. (2020). Phage therapy as a potential solution in the fight against AMR: Obstacles and possible futures. Palgrave Commun..

[12] Armstrong A.W., Mehta M.D., Schupp C.W., Gondo G.C., Bell S.J., Griffiths C.E.M. (2021). Psoriasis Prevalence in Adults in the United States. JAMA Dermatol..

[13] Zapi-Colín L., Gutiérrez-González G., Rodríguez-Martínez S., Cancino-Diaz J., Méndez-Tenorio A., Pérez-Tapia S., Gómez-Chávez F., Cedillo-Peláez C., Cancino-Diaz M. (2020). A peptide derived from phage-display limits psoriasis-like lesions in mice. Heliyon.

[14] Zhou X., Chen Y., Cui L., Shi Y., Guo C. (2022). Advances in the pathogenesis of psoriasis: From keratinocyte perspective. Cell Death Dis..

[15] Zhang X., Shi L., Sun T., Guo K., Geng S. (2021). Dysbiosis of gut microbiota and its correlation with dysregulation of cytokines in psoriasis patients. BMC Microbiol..

[16] Stehlikova Z., Kostovcik M., Kostovcikova K., Kverka M., Juzlova K., Rob F., Hercogova J., Bohac P., Pinto Y., Uzan A. (2019). Dysbiosis of Skin Microbiota in Psoriatic Patients: Co-occurrence of Fungal and Bacterial Communities. Front. Microbiol..

[17] Alekseyenko A.V., Perez-Perez G.I., De Souza A., Strober B., Gao Z., Bihan M., Li K., Methé B.A., Blaser M.J. (2013). Community differentiation of the cutaneous microbiota in psoriasis. Microbiome.

[18] Fahlén A., Engstrand L., Baker B.S., Powles A., Fry L. (2012). Comparison of bacterial microbiota in skin biopsies from normal and psoriatic skin. Arch. Dermatol. Res..

[19] Vieira A., Silva Y.J., Cunha A., Gomes N.C.M., Ackermann H.W., Almeida A. (2012). Phage therapy to control multidrug-resistant Pseudomonas aeruginosa skin infections: In vitro and ex vivo experiments. Eur. J. Clin. Microbiol. Infect. Dis. Off. Publ. Eur. Soc. Clin. Microbiol..

[20] Regeimbal J.M., Jacobs A.C., Corey B.W., Henry M.S., Thompson M.G., Pavlicek R.L., Quinones J., Hannah R.M., Ghebremedhin M., Crane N.J. (2016). Personalized Therapeutic Cocktail of Wild Environmental Phages Rescues Mice from Acinetobacter baumannii Wound Infections. Antimicrob. Agents Chemother..

[21] Ranjana W., Bharat J. (2021). Observational case studies of the effect of phage laden Ganga water on psoriasis. IP Indian J. Clin. Exp. Dermatol..

[22] Zambrano-Mila M.S., Blacio K.E.S., Vispo N.S. (2020). Peptide Phage Display: Molecular Principles and Biomedical Applications. Ther. Innov. Regul. Sci..

[23] Bhate K., Williams H.C. (2013). Epidemiology of acne vulgaris. Br. J. Dermatol..

[24] Golembo M., Puttagunta S., Rappo U., Weinstock E., Engelstein R., Gahali-Sass I., Moses A., Kario E., Cohen E.B., Nicenboim J. (2022). Development of a topical bacteriophage gel targeting Cutibacterium acnes for acne prone skin and results of a phase 1 cosmetic randomized clinical trial. Skin Health Dis..

[25] Mian M., Silfvast-Kaiser A., Paek S., Kivelevitch D., Menter A. (2019). A Review of the Most Common Dermatologic Conditions and their Debilitating Psychosocial Impacts. Int. Arch. Intern. Med..

[26] Dreno B., Martin R., Moyal D., Henley J.B., Khammari A., Seité S. (2017). Skin microbiome and acne vulgaris: Staphylococcus, a new actor in acne. Exp. Dermatol..

[27] Cavallo I., Sivori F., Truglio M., De Maio F., Lucantoni F., Cardinali G., Pontone M., Bernardi T., Sanguinetti M., Capitanio B. (2022). Skin dysbiosis and Cutibacterium acnes biofilm in inflammatory acne lesions of adolescents. Sci. Rep..

[28] Barnard E., Shi B., Kang D., Craft N., Li H. (2016). The balance of metagenomic elements shapes the skin microbiome in acne and health. Sci. Rep..

[29] Marinelli L.J., Fitz-Gibbon S., Hayes C., Bowman C., Inkeles M., Loncaric A., Russell D.A., Jacobs-Sera D., Cokus S., Pellegrini M. (2012). Propionibacterium acnes bacteriophages display limited genetic diversity and broad killing activity against bacterial skin isolates. mBio.

[30] Brüggemann H., Lood R. (2013). Bacteriophages infecting Propionibacterium acnes. BioMed Res. Int..

[31] Kim M.J., Eun D.H., Kim S.M., Kim J., Lee W.J. (2019). Efficacy of Bacteriophages in Propionibacterium acnes-Induced Inflammation in Mice. Ann. Dermatol..

[32] Lam H.Y., Lai M.J., Chen T.Y., Wu W.J., Peng S.Y., Chang K.C. (2021). Therapeutic Effect of a Newly Isolated Lytic Bacteriophage against Multi-Drug-Resistant Cutibacterium acnes Infection in Mice. Int. J. Mol. Sci..

[33] Brown T.L., Petrovski S., Dyson Z.A., Seviour R., Tucci J. (2016). The Formulation of Bacteriophage in a Semi Solid Preparation for Control of Propionibacterium acnes Growth. PLoS ONE.

[34] Thomsen S.F. (2014). Atopic Dermatitis: Natural History, Diagnosis, and Treatment. ISRN Allergy.

[35] Geoghegan J.A., Irvine A.D., Foster T.J. (2018). Staphylococcus aureus and Atopic Dermatitis: A Complex and Evolving Relationship. Trends Microbiol..

[36] Shimamori Y., Mitsunaka S., Yamashita H., Suzuki T., Kitao T., Kubori T., Nagai H., Takeda S., Ando H. (2020). Staphylococcal Phage in Combination with Staphylococcus epidermidis as a Potential Treatment for Staphylococcus aureus-Associated Atopic Dermatitis and Suppressor of Phage-Resistant Mutants. Viruses.

[37] Loc-Carrillo C., Abedon S.T. (2011). Pros and cons of phage therapy. Bacteriophage.

[38] Ly-Chatain M.H. (2014). The factors affecting effectiveness of treatment in phages therapy. Front. Microbiol..

[39] Duyvejonck H., Merabishvili M., Vaneechoutte M., de Soir S., Wright R., Friman V.-P., Verbeken G., De Vos D., Pirnay J.-P., Van Mechelen E. (2021). Evaluation of the Stability of Bacteriophages in Different Solutions Suitable for the Production of Magistral Preparations in Belgium. Viruses.

[40] Merabishvili M., Monserez R., van Belleghem J., Rose T., Jennes S., De Vos D., Verbeken G., Vaneechoutte M., Pirnay J.-P. (2017). Stability of bacteriophages in burn wound care products. PLoS ONE.

[41] Oechslin F. (2018). Resistance Development to Bacteriophages Occurring during Bacteriophage Therapy. Viruses.

[42] Yang Y., Shen W., Zhong Q., Chen Q., He X., Baker J., Xiong K., Jin X., Wang J., Hu F. (2020). Development of a Bacteriophage Cocktail to Constrain the Emergence of Phage-Resistant Pseudomonas aeruginosa. Front. Microbiol..

